# Facile synthesis of ternary graphene nanocomposites with doped metal oxide and conductive polymers as electrode materials for high performance supercapacitors

**DOI:** 10.1038/s41598-019-41939-y

**Published:** 2019-04-12

**Authors:** Saira Ishaq, Mahmoud Moussa, Farah Kanwal, Muhammad Ehsan, Muhammad Saleem, Truc Ngo Van, Dusan Losic

**Affiliations:** 1 Institute of Chemistry, University of the Punjab, Lahore, 54590 Pakistan; 2 School of Chemical Engineering, University of Adelaide, Adelaide, 5005 SA Australia; 3 ARC Research Hub for Graphene Enabled Industry Transformation, The University of Adelaide, Adelaide, 5005 SA Australia

## Abstract

Supercapacitors (SCs) due to their high energy density, fast charge storage and energy transfer, long charge discharge curves and low costs are very attractive for designing new generation of energy storage devices. In this work we present a simple and scalable synthetic approach to engineer ternary composite as electrode material based on combination of graphene with doped metal oxides (iron oxide) and conductive polymer (polypyrrole) with aims to achieve supercapacitors with very high gravimetric and areal capacitances. In the first step a binary composite with graphene mixed with doped iron oxide (rGO/MeFe_2_O_4_) (Me = Mn, Ni) was synthesized using new single step process with NaOH acting as a coprecipitation and GO reducing agent. This rGO/MnFe_2_O_4_ composite electrode showed gravimetric capacitance of 147 Fg^−1^ and areal capacitance of 232 mFcm^−2^ at scan rate of 5 mVs^−1^. In the final step a conductive polypyrrole was included to prepare a ternary composite graphene/metal doped iron oxide/polypyrrole (rGO/MnFe_2_O_4_/Ppy) electrode. Ternary composite electrode showed significantly improved gravimetric capacitance and areal capacitance of 232 Fg^−1^ and 395 mFcm^−2^ respectively indicating synergistic impact of Ppy additives. The method is promising to fabricate advanced electrode materials for high performing supercapacitors combining graphene, doped iron oxide and conductive polymers.

## Introduction

Energy consumption is continuously increasing throughout the world with 575 quadrillion British thermal units (BTU) in 2015 that is expected to rise up to 736 quadrillion BTU in 2040^1^. The current energy resources are insufficient to meet these energy demands which could cause serious energy crises in near future due to projected consumption of natural fuels, concerning greenhouse effect and heavily increased energy demands^2,3^. So there is an enormous and urgent need towards developing more efficient, low cost, easy to manufacture and environmental friendly energy solutions such as energy storage devices like fuel cells, batteries, capacitors and supercapacitors^4–7^. Among all energy storage devices, supercapacitors have advantages of long life cycles, rapid charging and discharging, high power density, rapid charge storage process and high energy density. Owing to these characteristics, supercapacitors are considered as complement of fuel cells, conventional rechargeable batteries and capacitors. Since the development of first commercial supercapacitor by Nippon Electric Company (NEC)^8^, supercapacitors have found profound applications across many sectors such as transportation, electronics, military, aerospace and sensors etc.

Carbon materials including activated carbon, carbon nanotubes (CNTs) and graphene have been extensively explored for supercapacitors applications even in general they display lower energy density due to the fast ions adsorption reaction thus generating electric double-layer (EDL) capacitances. On the other hand, metal oxides and conducting polymers can deliver much higher energy densities through Faradic reactions with low cyclic stability and power density compared to EDL based supercapacitors. Therefore combining these materials into their composite structure and using different charging mechanisms and possible synergistic effect between each of their components is recognized to be ideal solution to design and improve the performance of supercapacitors^9^.

Graphene has emerged as an ideal material for EDLCs due to its unique properties like high electrical conductivity, low density, high specific surface area (2670 m^2^ g^−1^), chemical stability, mechanical strength and tailoring chemical functionalities^10,11^. Initial studies showed gravimetric capacitance of synthesised graphene in aqueous and organic electrolytes to be 135 Fg^−1^ and 99 Fg^−1^ respectively^12^. Wang *et al*., prepared graphene by using gas based hydrazine reduction of graphene oxide (GO) and measured its gravimetric capacitance as 205 Fg^−1^^ 13^. Many other studies showed different results for gravimetric capacitance of graphene from 59 Fg^−1^ at scan rate of 2 mVs^−1^^ 14^, 117 Fg^−1^ at scan rate of 100 mVs^−1^ and 169.3 Fg^−1^ at 10 mVs^−1^ depending on the type of graphene, its purity and electrolyte^15^. Metal ferrites having variable redox states have been extensively explored as suitable electrode materials for supercapacitors applications^16,17^. Vignesh *et al*., used manganese ferrite supercapacitor electrode with specific capacitance of 173, 31 and 403 Fg^−1^ in 3.5 M KOH, 1 M LiNO_3_ and 1 M Li_3_PO_4,_ respectively measured by three electrodes system^18^. Aparna *et al*., reported comparative studies of various metal ferrites including Fe, Co, Ni, Mn, Cu and Zn in 3 M KOH solution used as electrolyte. Specific capacitance of metal ferrites (MeFe_2_O_4,_ Me = Fe, Co, Ni, Mn, Cu and Zn) were measured to be 101, 444.78, 109.26, 190, 250 and 138.95 Fg^−1^ respectively at scan rate of 2 mVs^−1^^19^. The first combination with of metal ferrites and graphene is demonstrated by Wang *et al*., who fabricated copper ferrite attached on graphene electrodes for supercapacitors showing an outstanding gravimetric capacitance 576.6 Fg^−1^ at current density of 1 Ag^−1^ measured by three electrodes system^20^.

In recent years, conducting polymers in pseudocapacitors are heavily explored due to their high specific capacitance obtained through reversible redox reaction. Polypyrrole is one of conducting polymers that showed excellent high conductivity and high environmental and mechanical stability^21,22^. Recently composites of polymers and nanofillers such as carbon based materials have been successfully used as electrodes to improve performance of supercapacitors using high synergistic effect. Biswas *et al*., synthesized graphene/polypyrrole composite material displaying gravimetric capacitance of 165 Fg^−1^ at current density of 1 Ag^−1^ measured by two electrodes system while using 1 M NaCl aqueous solution as electrolyte^23^. Parl *et al*., used graphite/polypyrrole composite for supercapacitor electrodes showing gravimetric capacitance of 400 Fg^−1^ measured by three electrodes system^24^.

In order to gain advanced supercapacitors performances, the concept of the three–components or ternary system by combining these three components has been proposed. Chee *et al*., synthesized ternary polypyrrole/graphene oxide/zinc oxide supercapacitor electrodes and measured its gravimetric capacitance in two electrodes system to be 94.6 Fg^−1^ at 1 Ag^−1^ from charge/discharge (CD) curves^25^. Lim *et al*., reported ternary polypyrrole/graphene/nano manganese oxide composite, the gravimetric capacitance of synthesized composite was 320.6 Fg^−1^ at 1 mVs^−1^ which was much higher than that of polypyrrole/graphene delivering gravimetric capacitance of 255.1 Fg^−1^ and neat polypyrrole with gravimetric capacitance of 118.4 Fg^−1^^ 26^. Xiong *et al*., used three electrodes system to measure gravimetric capacitance of ternary cobalt ferrite/graphene/polyaniline nanocomposites, which showed gravimetric capacitance of 1133.3 Fg^−1^ at scan rate of 1 mVs^−1^^ 27^. These studies clearly indicate that design of multi-component composite electrodes for supercapacitors is a beneficial and promising approach that is able to significantly improve the performance of supercapacitors.

**Figure 1:**
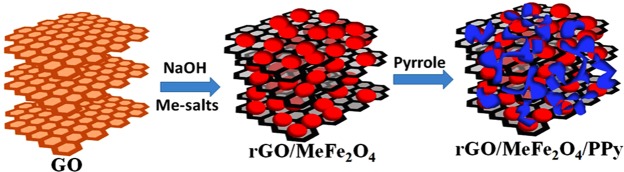
Schematics of synthesis of binary rGO/MeFe_2_O_4_ and ternary rGO/MeFe_2_O_4_/Ppy nanocomposite as new electrode material for supercapacitors.

Inspired with these studies in the present research work, we present the synthesis and performances of ternary composite electrodes for supercapacitor applications that are specifically engineered by combination of graphene, mixed doped metal oxide and conductive polymers and their unique properties and synergistic effects. To demonstrate this concept we selected graphene (rGO), metals doped iron oxide (MeFe_2_O_4_) and conductive polypyrrole (Ppy) polymer as model components for proposed ternary system (rGO/MeFe_2_O_4_/Ppy) that is schematically presented in Fig. 1. The aims of this work were to explore the electrochemical performance of this ternary composite material, evaluate influence of each component and their synergetic effects and demonstrate its capability to be used for designing high performing supercapacitors. For metal doping of iron oxide two metals Mn and Ni were selected as a model doping elements because of their excellent redox behaviour. These metals can contribute more efficiently than pure iron oxide in increasing gravimetric capacitance having high electronic conductivity and electrochemical performance, low cost and environmental friendly nature^28^. Finally Ppy was selected as common and highly conductive polymer having high specific capacitance of 136 Fg^−1^ measured by three electrodes system^29^. In addition to confirm the performance of proposed ternary electrodes system one of specific objective of this work was to develop new, simplified, environmentally friendly and scalable method to synthesize these composite materials. For that purpose we introduced one step process using NaOH to make binary composite (rGO/MeFe_2_O_4_) instead of undergoing conventional two steps process using reduction by hydrazine hydrate to form reduced graphene oxide (rGO). In the following and final step ternary graphene/metal doped iron oxide/polypyrrole (rGO/MeFe_2_O_4_/Ppy) nanocomposite was synthesized by common oxidative polymerization of pyrrole. The extensive characterization for both binary and ternary composites with mixed metals (Mn and Ni) as dopant of iron oxide was performed in order to reveal synergetic impact of each component in the system on supercapacitor performances.

## Results and Discussion

### Characterization of prepared binary graphene/metal doped iron oxide (rGO/MeFe_2_O_4_) and ternary graphene/metal doped iron oxide/polypyrrole (rGO/MeFe_2_O_4_/Ppy) nanocomposites

**Figure 2:**
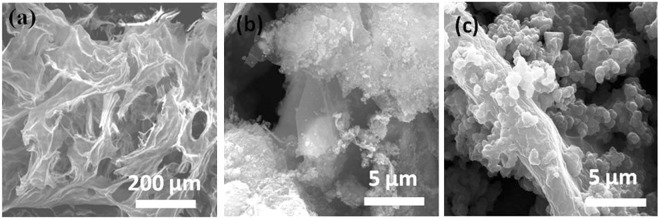
Comparative FESEM images of (**a**) GO (**b**) binary rGO/MnFe_2_O_4_ and **(c**) ternary rGO/MnFe_2_O_4_/Ppy composite structure.

Morphologies of prepared binary rGO/MnFe_2_O_4_ and ternary rGO/MnFe_2_O_4_/Ppy composites are summarised in field emission scanning electron microscopy (FESEM) images presented in Fig. 2. FESEM image of rGO/MnFe_2_O_4_ reveals sponge like porous structure showing the MnFe_2_O_4_ nano rods distributed on graphene sheets^30^. Average size of MnFe_2_O_4_ nanorods was found to be in the range of 50–70 nm. The comparison of morphology of binary rGO/MnFe_2_O_4_ and ternary rGO/MnFe_2_O_4_/Ppy composite at various magnifications is presented in FESEM images in Fig. S1 (supporting information). FESEM image of rGO/NiFe_2_O_4_ and rGO/NiFe_2_O_4_/Ppy shown in Fig. S2A also confirms formation of rGO sheets on which NiFe_2_O_4_ nanorods are randomly distributed. Ppy is also visible in the FESEM images of both ternary rGO/MnFe_2_O_4_/Ppy (Fig. 2c) and rGO/NiFe_2_O_4_/Ppy nanocomposites (Fig. S2).

**Figure 3:**
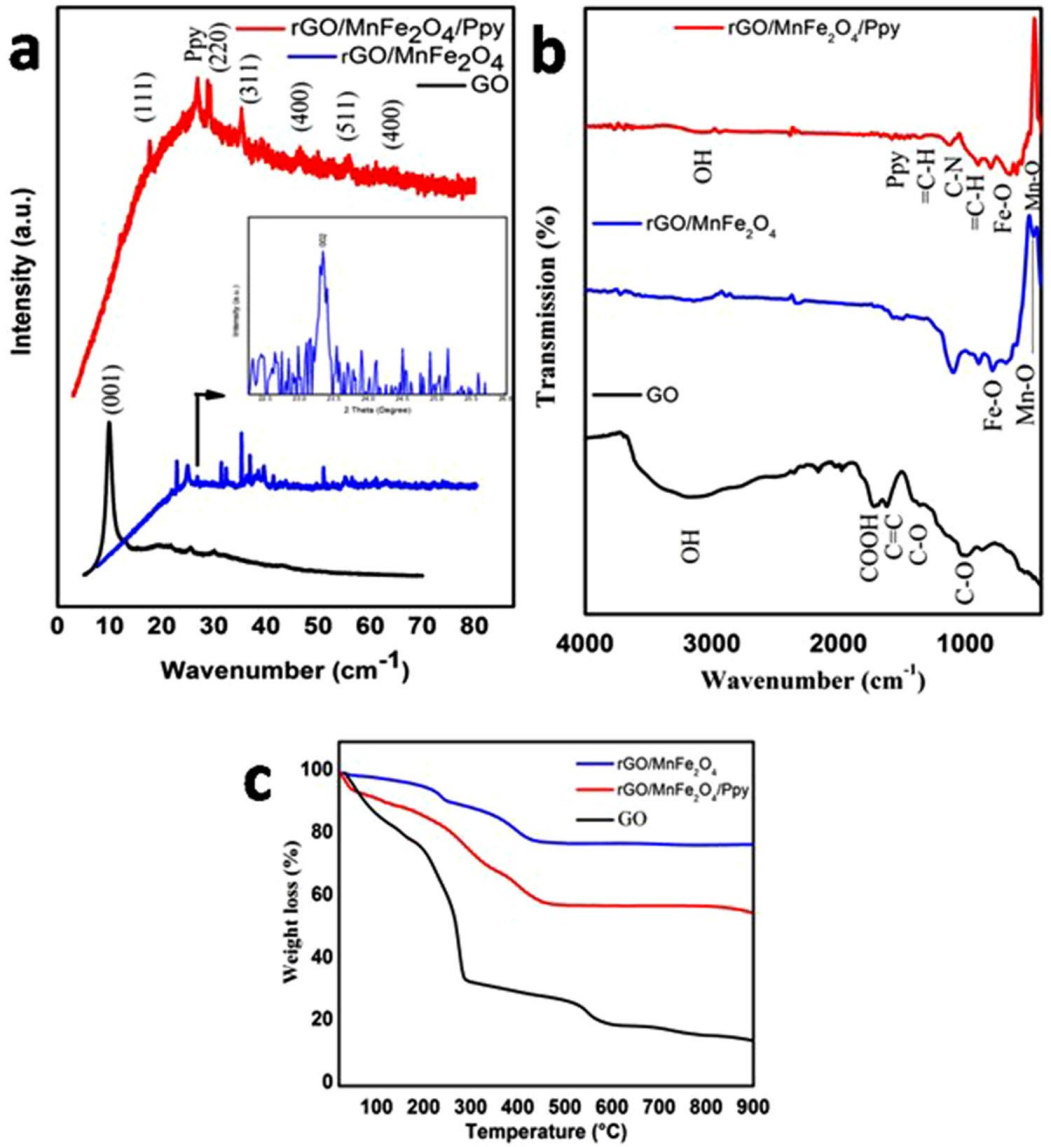
Comparative characterization of GO, rGO/MnFe_2_O_4_ and rGO/MnFe_2_O_4_/Ppy electrode during different stage of composite preparations showing their (**a**) XRD pattern of GO, rGO/MnFe_2_O_4_ and rGO/MnFe_2_O_4_/Ppy nanocomposite (**b**) FTIR spectra, (**c**) TGA graphs.

Comparative crystal structure characterization of GO, rGO/MnFe_2_O_4_ and rGO/MnFe_2_O_4_/Ppy was carried out by XRD showing their XRD patterns in Fig. 3a. Characteristic peak of GO appearing at 10.9° (001) in XRD pattern of pure GO disappears in the XRD patterns of composites which shows complete oxidation of graphite into graphene oxide (GO)^31^. In XRD pattern of rGO/MnFe_2_O_4_ peaks at 19.2°, 29.1°, 35.2°, 41.1°, 57° and 61.9° indexed to (111), (220), (311), (400), (511) and (440) respectively are in accordance to JCPDS card no. 73–1964 and confirm formation of MnFe_2_O_4_^32^. Peak for rGO at 25° (002) was observed in rGO/MnFe_2_O_4._ It confirms reduction of GO into rGO^33^. However this peak is not very sharp due to destruction of regular stacking of graphene sheets. XRD pattern of NiFe_2_O_4_ shown in Fig. S2B also confirms reduction of GO into rGO. XRD patterns of as synthesized rGO/MnFe_2_O_4_/Ppy and rGO/NiFe_2_O_4_/Ppy nanocomposites in Fig. 3a and S2B show characteristic peak of Ppy at about 2θ = 26°. This characteristic broad peak shows that Ppy is in amorphous form^34^. Along with peak of Ppy, other peaks for rGO, rGO/MnFe_2_O_4_ and rGO/NiFe_2_O_4_ nanocomposites also appear. Some other peaks are also present showing impurities in the sample.

Comparative FTIR spectra of GO, rGO/MnFe_2_O_4_ and rGO/MnFe_2_O_4_/Ppy are summarized in Fig. 3b. FTIR spectrum of GO shows peaks at 3410 cm^−1^ and 1730 cm^−1^ corresponding to stretching vibrations of hydroxyl (OH) and carboxyl (COOH) groups respectively. Peak observed at 1604 cm^−1^ confirms presence of aromatic C = C which is an indication of hybrid sp^2^ structure of rGO. Peak at 1420 cm^−1^ shows presence of carboxy (C-O) group. Peaks at 1050 cm^−1^ and 1220 cm^−1^ are due to presence of epoxy and alkoxy/alkoxide C-O groups respectively^35^. FTIR spectrum of rGO/MnFe_2_O_4_ shows peaks of both rGO and MnFe_2_O_4_ confirming formation of rGO/MnFe_2_O_4._ Peaks at 460.99 cm^−1^ and 569.0 cm^−1^ are related to Mn-O and Fe-O respectively^36^. A peak observed at 1604 cm^−1^ for GO indicating C = C skeletal vibration of un-oxidized graphitic domains was red-shifted to 1530 cm^−1^ showing that aromatic vibration is still present in rGO. Other peaks of GO related to oxygen functional groups do not appear in FTIR spectrum of rGO/MnFe_2_O_4_ showing conversion of GO into rGO during reduction process^37,38^. FTIR spectrum GO/NiFe_2_O_4_ and rGO/NiFe_2_O_4_/Ppy shown in Fig. S2C also show all these peaks thus confirm formation of other graphene metal doped iron oxide nanocomposites i.e., rGO/NiFe_2_O_4._ Both rGO/MnFe_2_O_4_/Ppy and rGO/NiFe_2_O_4_/Ppy show peaks for rGO, metal ferrites and Ppy confirming formation of ternary rGO/MnFe_2_O_4_/Ppy and rGO/NiFe_2_O_4_/Ppy nanocomposites. However peaks for metal ferrite nanoparticles are weak due to their uniform distribution on Ppy backbone. In addition to the peaks of metal ferrites below 700 cm^−1^, peaks of Ppy appear like fundamental vibrations of Ppy ring at 1556 and 1449 cm^−1^, =C-H in-plane vibrations at 1303 cm^−1^ and 1036 cm^−1^, C-N stretching vibrations at 1196 cm^−1^, polymerized pyrrole at 796 cm^−1^ and 983 cm^−1^, = C-H out of plane vibration at 903 cm^−1^, and OH and oxide groups at 3400 cm^−1^^ 39^. These peaks are shifted left than peaks of pure Ppy which indicates that graphene groups are associated to nitrogenous functional groups of Ppy like normal doping process to the Ppy^40^.

The TGA characterizations carried out to study thermal stability of GO, rGO/MeFe_2_O_4_ and rGO/MeFe_2_O_4_/Ppy nanocomposites is presented in Fig. 3c and S2D. TG curves of GO and rGO/MnFe_2_O_4_ in Fig. 3c shows that a slight weight loss below 200 °C was observed for GO and rGO/MnFe_2_O_4_ and it is due to water loss. For GO maximum weight is lost below 300 °C. This weight loss is due to break down of oxygen functional groups in GO. However it is apparent from TG curve of rGO/MnFe_2_O_4_ that it is thermally more stable than GO. It is due to conversion of GO into rGO, which is due to removal of oxygen functional groups during reduction of GO to rGO^41^. Same effect is observed for rGO/NiFe_2_O_4._ Slight weight loss of rGO/MeFe_2_O_4_ was observed between 230–450 °C. No noticeable change was observed after 420 °C and residual weight was about 77% and 35% for rGO/MnFe_2_O_4_ and rGO/NiFe_2_O_4_ respectively (Figs 3c and S2D), which can be ascribed to remaining MnFe_2_O_4_ in rGO/MnFe_2_O_4_ and NiFe_2_O_4_ in rGO/NiFe_2_O_4_ respectively. rGO/MeFe_2_O_4_/Ppy show more weight loss than rGO/MeFe_2_O_4_ which shows that thermal stability of rGO/MeFe_2_O_4_ is more than that of rGO/MeFe_2_O_4_/Ppy. This trend is similar in all nanocomposites and is in accordance with results depicted in previous literature^42^. Residual weight of rGO/MnFe_2_O_4_/Ppy and rGO/NiFe_2_O_4_/Ppy is 55% and 13% which is 22% less than their binary composites i.e., rGO/MnFe_2_O_4_ and rGO/NiFe_2_O_4._This decrease in thermal stability of rGO/MeFe_2_O_4_/Ppy is due to Ppy addition. Due to increase in ion mobility together with thermal vibration of Ppy chains at rGO/MeFe_2_O_4_ and Ppy interface, thermal stability of rGO/MeFe_2_O_4_/Ppy is less than that of rGO/MeFe_2_O_4_^43^.

### Evaluation of electrochemical and supercapacitor performance of binary graphene/metal doped iron oxide (rGO/MeFe_2_O_4_) nanocomposites

Electrochemical characterizations of prepared nanocomposites was performed in two stages by testing electrochemical performances of binary rGO/MeFe_2_O_4_ nanocomposite and their individual components followed by characterization of final ternary nanocomposites in order to evaluate influence of each components in composite and their synergistic impact.

**Figure 4:**
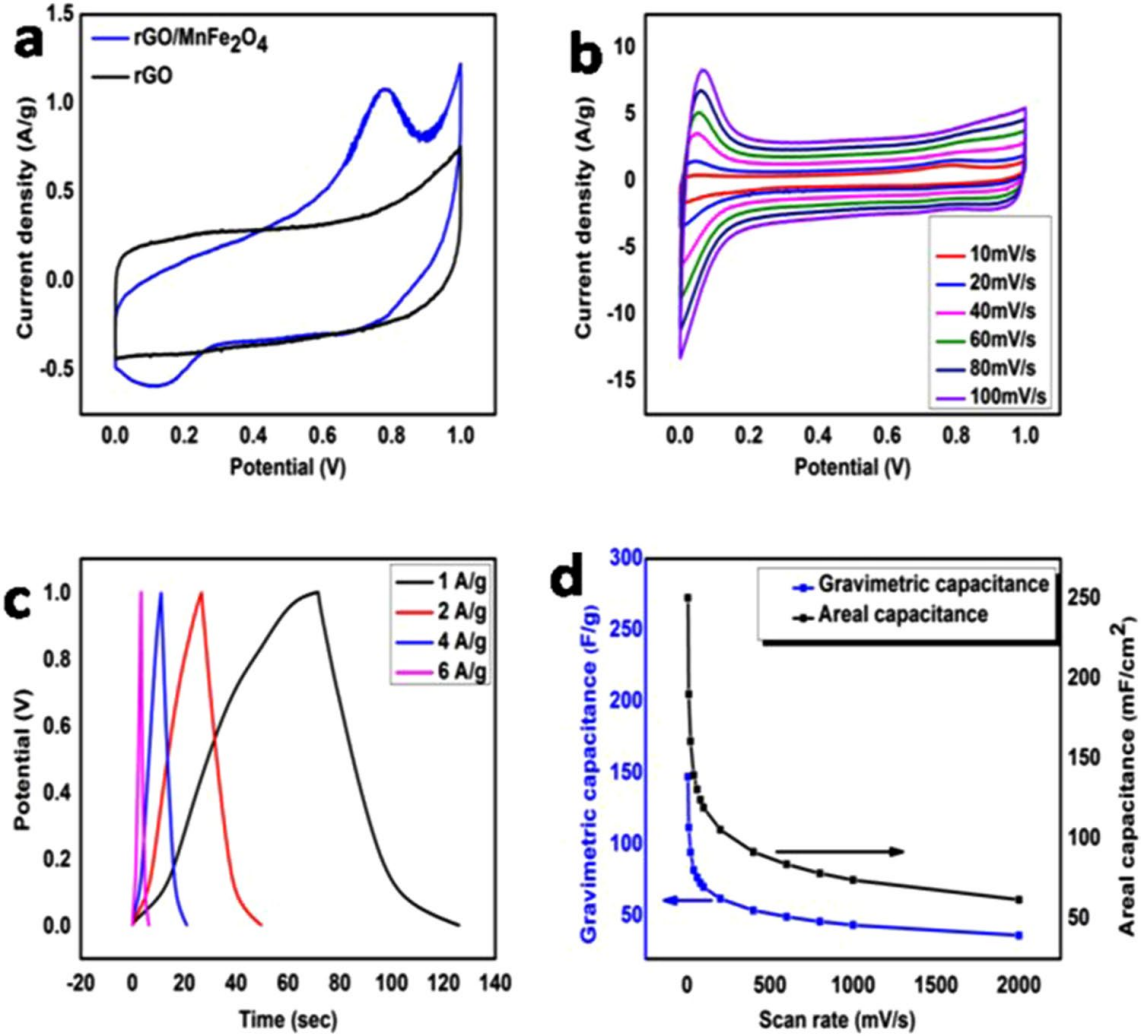
(**a**) CV curves of rGO and rGO/MnFe_2_O_4_ nanocomposite at 5 mVs^−1^. (**b**) CV curves of rGO/MnFe_2_O_4_ nanocomposite at different scan rates (10–100mVs^−1^). (**c**) CD curves of rGO/MnFe_2_O_4_ at different current density (1 Ag^−1^, 2 Ag^−1^, 4 Ag^−1^ and 6 Ag^−1^). (**d**) Gravimetric and areal capacitances of rGO/MnFe_2_O_4_ at different scan rates (10–2000mVs^−1^).

CV curve of graphene present in Figs 4a and S3A are almost rectangular, showing no oxidation and reduction peaks. It is characteristic of carbon based materials showing EDLC^44^. Redox peaks appear in CV curves of rGO/MnFe_2_O_4_ and rGO/NiFe_2_O_4_ shown in Figs 3a and S3A, respectively. These redox peaks are due to Faradic process. These redox peaks indicate pseudocapacitance behaviour of MeFe_2_O_4_ in the nanocomposite. The redox process is described  in equations 1–2^44^.

1 $${{\rm{MnFe}}}_{2}{{\rm{O}}}_{4}+{{\rm{H}}}_{2}{\rm{O}}+{{\rm{OH}}}^{-}\leftarrow ----\to {\rm{MnOOH}}+2{\rm{FeOOH}}+{{\rm{e}}}^{-}$$

2 $${\rm{FeOOH}}+{{\rm{OH}}}^{-}\leftarrow ------\to {{\rm{FeO}}}_{2}+{{\rm{H}}}_{2}{\rm{O}}+{{\rm{e}}}^{-}$$

Figures 4b and S3B show the CV curves of rGO/MnFe_2_O_4_ and rGO/NiFe_2_O_4_ respectively at different scan rates of 10–100 mVs^−1^. It is clear from the figures that current density of peak enhances with corresponding scan rate. It depicts its fairly good ion response and good EDL capacitance behaviour. High current density with increasing scan rate depicts that electrode material shows more conductivity, less internal resistance and good rate capability of electrode material in used electrolyte i.e., 1 M H_2_SO_4_ as the scan rate increases^44^. Shape of CV curves remains same at different scan rates of 10–100 mVs^−1^ which shows that kinetics of EDL formation is quite fast and it is also indicative of fast Faradic reaction in electrodes^45^. Gravimetric capacitance of rGO/MnFe_2_O_4_ electrode was 147 Fg^−1^ at scan rate of 5 mVs^−1^. At the same scan rate, areal capacitance of rGO/MnFe_2_O_4_ electrode calculated from its CV curves was 250 mFcm^−2^ while gravimetric and areal capacitance of rGO/NiFe_2_O_4_ electrode was found to be 48 Fg^−1^ and 82 mFcm^−2^, respectively.

CD curves of rGO/MnFe_2_O_4_ at different current densities of 1 Ag^−1^, 2 Ag^−1^, 4 Ag^−1^ and 6 Ag^−1^ are shown in Fig. 4c. CD curves of synthesized rGO/MnFe_2_O_4_ based electrodes are not symmetrical, which shows pseudocapacitive behaviour of electrode material. CD curves at different current densities are of almost same shape, showing that electrode material has ideal capacitive behaviour. Similar behaviour was observed in CD curves of rGO/NiFe_2_O_4_ as shown in Fig. S3C.

Figure 4d represents gravimetric and areal capacitances of rGO/MnFe_2_O_4_ at different scan rates i.e., 10–2000 mVs^−1^. It is evident that with increase in scan rate both gravimetric capacitance and areal capacitance decrease. It is attributed to insufficient time available for ions to diffuse and adsorb in the small pores within large particles. In addition, at high scan rate, when supercapacitor delivers high current, a noticeable voltage loss (ΔV) is originated^46^.

### Evaluation of Electrochemical and supercapacitor performance of ternary graphene/metal doped iron oxide/polypyrrole (rGO/MeFe_2_O_4_/Ppy) nanocomposites

**Figure 5:**
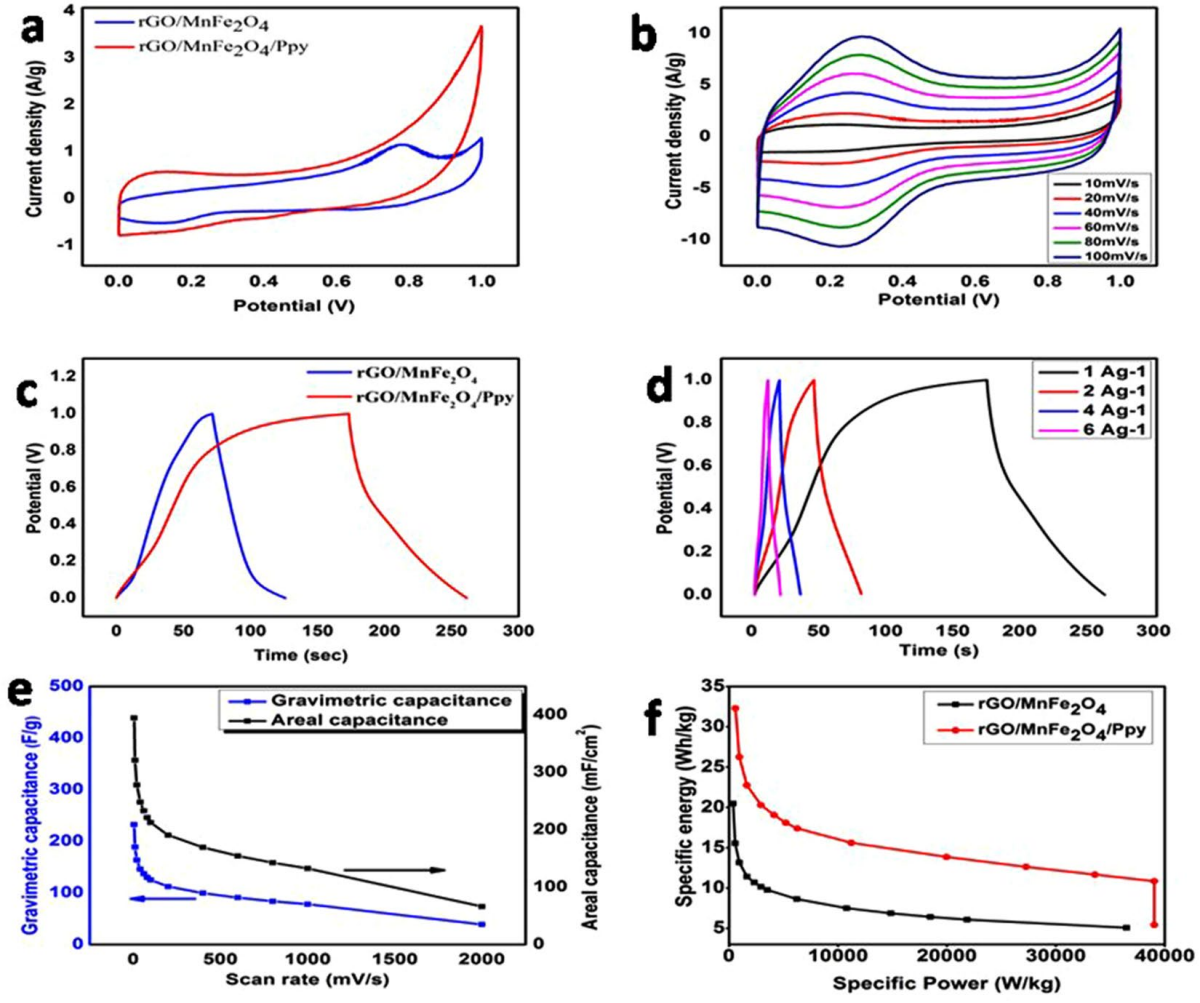
(**a**) CV curves of rGO/MnFe_2_O_4_ and rGO/MnFe_2_O_4_/Ppy at 5 mVs^−1^. (**b**) CV curves of rGO/MnFe_2_O_4_/Ppy nanocomposite at different scan rates (10–100mVs^−1^). (**c**) CD curves of rGO/MnFe_2_O_4_ and rGO/MnFe_2_O_4_/Ppy at 1Ag^−1^. (**d**) CD curves of rGO/MnFe_2_O_4_/Ppy at different current density 1 Ag^−1^, 2 Ag^−1^, 4 Ag^−1^ and 6 Ag^−1^. (**e**) Gravimetric and Areal capacitance of rGO/MnFe_2_O_4_/Ppy at different scan rates (5–2000 mVs^−1^) (**f**) Ragon plots of rGO/MnFe_2_O_4_ and rGO/MnFe_2_O_4_/Ppy at different scan rates (5–2000 mVs^−1^).

Figure 5a shows CV curves of rGO/MnFe_2_O_4_ and rGO/MnFe_2_O_4_/Ppy at 5 mVs^−1^. It is evident that CV curve area of rGO/MnFe_2_O_4_/Ppy is larger than curve area of rGO/MnFe_2_O_4_ showing better capacitance of former than later. Similar results are shown in Fig. S4A for rGO/NiFe_2_O_4_ and rGO/NiFe_2_O_4_/Ppy. CV curves of rGO/MnFe_2_O_4_/Ppy and rGO/NiFe_2_O_4_/Ppy at different scan rates of 10–100 mVs^−1^ are shown in Figs 5b and S4B, respectively. Redox peaks appear in CV curves of both rGO/MnFe_2_O_4_/Ppy and rGO/NiFe_2_O_4_/Ppy. These redox peaks are attributed to pesudocapacitance of MeFe_2_O_4_ in the nanocomposite. rGO/MnFe_2_O_4_/Ppy shows largest current density at same scan rate among all electrodes. Owing to this large current density and symmetrical behaviour of CV curves in both anodic and cathodic directions it is proposed to possess best capacitive performance among all synthesized electrodes and it is suitable supercapacitor electrode material^47^. Gravimetric capacitance of rGO/MnFe_2_O_4_/Ppy electrode was 232 Fg^−1^ at scan rate of 5 mVs^−1^ while its areal capacitance was 395 mFcm^−2^. It is obvious that both gravimetric and areal capacitance of rGO/MnFe_2_O_4_/Ppy was 1.57 times greater than that of rGO/MnFe_2_O_4_ due to more synergistic effect between the composite components_._ CV curves of both rGO/MnFe_2_O_4_/Ppy and rGO/NiFe_2_O_4_/Ppy are rectangular even at high scan rate of 100 mVs^−1^ showing that this electrode material has higher electrochemical performance^48^. High current density with increasing scan rate depicts that electrode material shows more conductivity, less internal resistance and good rate capability of electrode material in used electrolyte i.e., 1 M H_2_SO_4_ as the scan rate increases^44^. Similar trend was observed in rGO/NiFe_2_O_4_/Ppy electrode shown in Fig. S4B.

Figure 5c shows CD curve of rGO/MnFe_2_O_4_ and rGO/MnFe_2_O_4_/Ppy showing consistent results with those of cyclic voltammetry. It is clear that discharge time for rGO/MnFe_2_O_4_/Ppy is much greater than that of rGO/MnFe_2_O_4._ Moreover, CD curve for rGO/MnFe_2_O_4_/Ppy is less symmetrical than that of rGO/MnFe_2_O_4_ showing that after addition of Ppy, the pesudocapacitance of material is enhanced^49^. This increase in electrochemical performance of rGO/MnFe_2_O_4_/Ppy, as depicted by cyclic voltammetry and charge discharge measurements, is due to synergistic effect of three components in ternary rGO/MnFe_2_O_4_/Ppy nanocomposite. CD curves of rGO/MnFe_2_O_4_/Ppy at different current densities of 1 Ag^−1^, 2 Ag^−1^, 4 Ag^−1^ and 6 Ag^−1^ are shown in Fig. 5d. All CD curves are of almost same shape at all current densities, showing that electrode material has ideal capacitive behaviour. Among all synthesized electrodes, discharge time of rGO/MnFe_2_O_4_/Ppy electrode is maximum showing its best electrochemical performance. CD curves of rGO/NiFe_2_O_4_ and rGO/NiFe_2_O_4_/Ppy electrodes are shown in Fig. S4C,D have similar trends.

Results of cyclic voltammetry and charge discharge measurements are summarized in Fig. 5e in the form of gravimetric capacitance and areal capacitance of rGO/MnFe_2_O_4_ and rGO/MnFe_2_O_4_/Ppy at various scan rates i.e., 5–2000 mVs^−1^. Improved electrochemical performance of material after addition of Ppy can be explained by considering conductive nature of Ppy which results in more diffusion of ions into electroactive electrode material as well as synergistic effect of components^50^. For rGO/NiFe_2_O_4_ and rGO/NiFe_2_O_4_/Ppy, gravimetric and areal capacitances at different scan rates of 5–2000 mVs^−1^ are summarized in Fig. S4E.

Specific power and specific energy of rGO/MnFe_2_O_4_ and rGO/MnFe_2_O_4_/Ppy at different scan rates of 5–2000 mVs^−1^ were calculated. Ragon plots of rGO/MnFe_2_O_4_ and rGO/MnFe_2_O_4_/Ppy are shown in Fig. 5f. At 5 mVs^−1^ specific power of rGO/MnFe_2_O_4_ and rGO/MnFe_2_O_4_/Ppy is is 368.5 Wkg^−1^ and 581 Wkg^−1^, respectively which is increased to 36479.5 Wkg^−1^ and 39034 Wkg^−1^ at 2000 mVs^−1^. While specific energy values of rGO/MnFe_2_O_4_ and rGO/MnFe_2_O_4_/Ppy are 20.5 and 32.3 Whkg^−1^, respectively at 5 mVs^−1^. It decreases to 5.1 Whkg^−1^ and 5.4 Whkg^−1^, respectively. Increases in specific energy from rGO/MnFe_2_O_4_ to rGO/MnFe_2_O_4_/Ppy is due to increased electrical conductivity due to addition of Ppy in rGO/MnFe_2_O_4_/Ppy^51^. For rGO/NiFe_2_O_4_ and rGO/NiFe_2_O_4_/Ppy, specific power and specific energy at different scan rates of 5–2000 mVs^−1^ are summarized in Fig. S4F.

**Figure 6:**
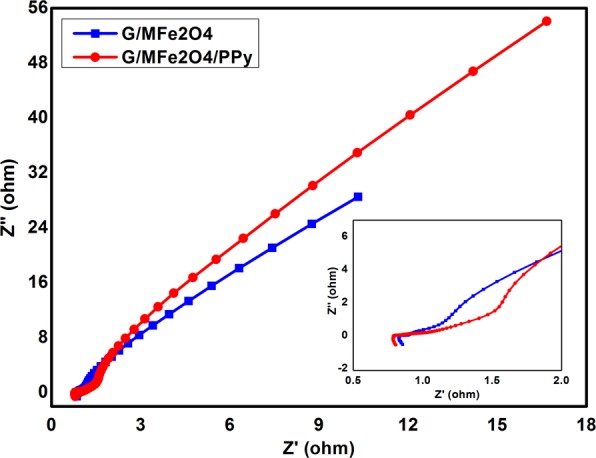
Nyquist plots of rGO/MnFe_2_O_4_ and rGO/MnFe_2_O_4_/Ppy.

**Figure 7:**
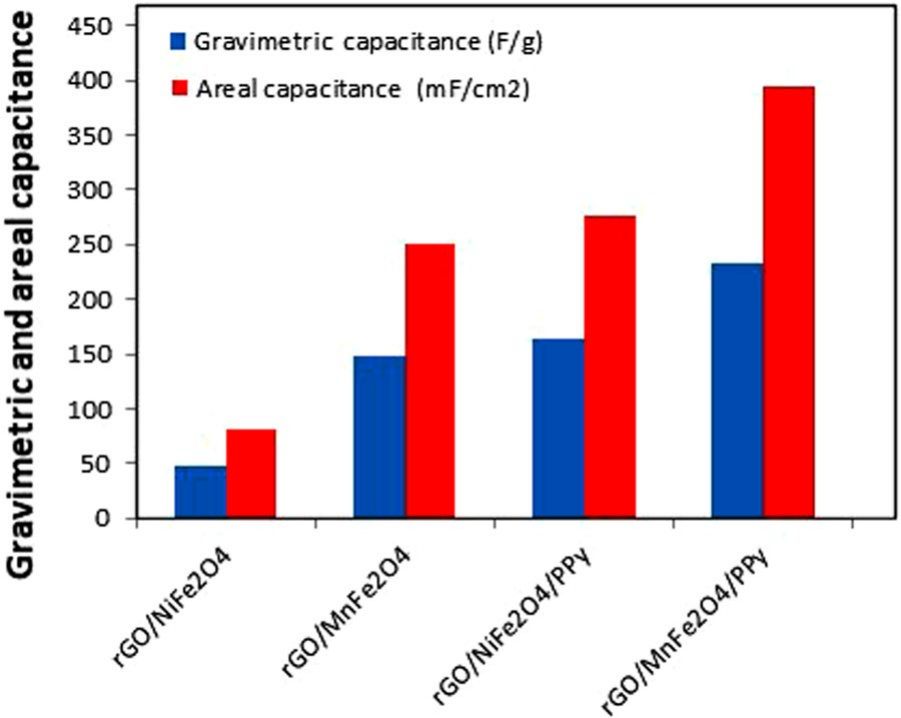
Comparative gravimetric capacitance and areal capacitance of rGO/MnFe_2_O_4,_ rGO/MnFe_2_O_4_/Ppy, rGO/NiFe_2_O_4_ and rGO/NiFe_2_O_4_/Ppy nanocomposites at 5 mVs^−1^.

Electrochemical Impedance spectroscopy (EIS) has been performed by two electrodes system in 1 M H_2_SO_4_ at the excited potential of 5 mV between frequency range of 0.01–100 kHz in the form of Nyquist plot shown in Fig. 6. Nyquist plot reveals that rGO/MnFe_2_O_4_ and rGO/MnFe_2_O_4_/Ppy show very small semicircles at high frequency region, which reveal to charge transfer resistance and solution resistance while a straight line in high frequency region reveals to Warburg resistance. rGO/MnFe_2_O_4_/Ppy shows small equivalent series resistance of 0.81 Ω while rGO/MnFe_2_O_4_ shows greater resistance of 0.86 Ω. This equivalent series resistance (ESR) was calculated from first x-intercept and slope of Nyquist plot^52^. However, in low frequency region rGO/MnFe_2_O_4_/Ppy has more slope as compared to slope of rGO/MnFe_2_O_4_ explaining that former has better capacitance than later that is consistent with results of CV and CD. Nyquist plots for rGO/NiFe_2_O_4 _and ternary rGO/NiFe_2_O_4_/Ppy nanocomposites are shown in Fig. S5. Figure 7 summarizes results of gravimetric and areal capacitance of all synthesized binary rGO/MeFe_2_O_4_ and ternary rGO/MeFe_2_O_4_/Ppy nanocomposites.

Table 1 shows comparison of the electrochemical performance of synthesized ternary rGO/MeFe_2_O_4_/Ppy electrodes with electrochemical performances of previously reported electrodes. Comparison of our results with previous literature findings shows that ternary rGO/MnFe_2_O_4_/Ppy electrodes give better electrochemical performance compared with previous reports. We propose that possible reason of this improvement is the incorporation of MnFe_2_O_4_ particle as third component which improves electrochemical performance due to its redox behaviour. While gravimetric capacitance of rGO/NiFe_2_O_4_/Ppy is slightly less than that of G/Ppy showing that NiFe_2_O_4_ contributes less gravimetric capacitance than MnFe_2_O_4_. Gravimetric capacitance of rGO/NiFe_2_O_4_/Ppy, rGO/MnFe_2_O_4_ and rGO/MnFe_2_O_4_/Ppy is better than previously reported Ppy/GO/ZnO composite electrode which may be due to reasons that rGO is more conducting than GO and MeFe_2_O_4_ is better candidate than ZnO to enhance electrochemical performance. CNT/Ppy/MnO_2_ electrodes deliver more gravimetric capacitance than our synthesized electrodes because in synthesis of this composite hydrous MnO_2_ was used to disperse it effectively in polymer matrix. Better gravimetric capacitance of previously reported PANI-Graphene-CNT electrodes as compared to our synthesized binary and ternary composites can be explained due to presence of all three highly conducting components, each of which may effectively participate in improving the electrochemical performance of resulting composite. Moreover, electrolyte used in that supercapacitor is different than used by us and their value was calculate at low current density i.e., 0.5 Ag^−1^. Usually at low current density gravimetric capacitance is high which decreases accordingly with increasing current density. Areal capacitance of previously reported PEDOT-NiFe_2_O_4_ electrode is almost equal to that of binary rGO/MnFe_2_O_4_ electrode. However it is less than our synthesized rGO/MeFe_2_O_4_/Ppy electrodes. It is due to presence of conducting Ppy in rGO/NiFe_2_O_4_/Ppy. Areal capacitance of our binary rGO/MnFe_2_O_4_ and ternary rGO/MeFe_2_O_4_/Ppy is better than previously reported PEDOT-GO/CNTs and Ppy-GO/CNTs which is due to more conductive rGO than GO used in above mentioned composites. Overall comparison suggests our synthesized ternary rGO/MeFe_2_O_4_/Ppy composite as suitable supercapacitor electrode material with efficient electrochemical performance.

**Table 1:** Comparison of the specific capacitances of ternary rGO/MeFe_2_O_4_/Ppy electrodes with specific capacitances of previously reported electrodes.

Electrode material	Galvanic discharge/Scan rate	Electrolyte	Gravimetric capacitance (Fg^−1^)	Areal capacitance (mFcm^−2^)	References
Ppy/GO/ZnO	1 Ag^−1^	1 M Na_2_SO_4_	94.6	—	^25^
CNT/Ppy/MnO_2_	20 mVs^−1^	1 M Na_2_SO_4_	281	—	^56^
PANI-Graphene-CNT	0.5 Ag^−1^	PVA-H_3_PO_4_	890	—	^57^
PEDOT-GO/CNTs	10 mVs^−1^	1 M KCl	—	104	^58^
Ppy-GO/CNTs	10 mVs^−1^	1 M KCl	—	143.6	^58^
rGO/NiFe_2_O_4_/Ppy	5 mVs^−1^	1 M H_2_SO_4_	162.9	276.9	This work
rGO/MnFe_2_O_4_/Ppy	5 mVs^−1^	1 M H_2_SO_4_	232.0	395.2	This work

## Materials and Methods

### Materials

Graphite powder was purchased from Merck and form local graphite company (Ulley, South Australia). Glycerine was purchased from Fischer Scientific. K_2_S_2_O_8_ was purchased from Scharlau span, Hydrazine monohydrate was purchased from Daejung. P_2_O_5_, MnSO_4_.H_2_O, FeSO_4_.7H_2_O and H_2_O_2_ were purchased from Riedel-deHaen. Pyrrole was purchased from Sigma Aldrich, NiCl_2_.6H_2_O was purchased from United Laboratory Chemicals. While FeCl_3_.6H_2_O and polytetrafluoroethylene were purchased from Aldrich. Commercial NaOH, HCl, H_2_SO_4_, Ethanol and acetone were used. All chemicals were pure and of analytical grade and were used without further purification.

### One step synthesis of binary graphene/metal doped iron oxide nanoparticles (rGO/MeFe_2_O_4_)

Graphene oxide (GO) was synthesized by a modified Hummer’s method^53^. Binary Graphene/manganese ferrite (rGO/MnFe_2_O_4_) was prepared by in-situ reduction coprecipitation method described previously in literature^54^. Briefly speaking, measured amount of GO was well dispersed into the distilled water by ultrasonication. Then MnSO_4_.H_2_O and FeSO_4_.7H_2_O (1:2) were added followed by vigorous stirring at 95 °C for 2 h under N_2_ protection and 1 h in air. Subsequently, 2 M NaOH solution was slowly added to the mixture to adjust the solution to pH of 11–12. After 2 h of stirring, the solution was cooled to room temperature (RT). Final precipitates were collected by centrifugation, washed with distilled water thrice and dried at 80 °C for 24 h to obtain binary rGO/MnFe_2_O_4_ nanocomposite.

Similarly binary graphene/nickel ferrite (rGO/NiFe_2_O_4_) nanocomposite was synthesized by using NiCl_2_.6H_2_O and FeCl_3_.6H_2_O (1:2) as raw materials, 2–3 drops of glycerine as surfactant, and NaOH as base as well as reducing agent.

### Synthesis of ternary graphene/metal ferrite/polypyrrole nanocomposites (rGO/MeFe_2_O_4_/Ppy)

To synthesize ternary graphene/metal ferrite/polypyrrole nanocomposites (rGO/MFe_2_O_4_/Ppy), polymerization method of pyrrole^55^ was adopted with modifications. Briefly describing, measured amount of rGO/MeFe_2_O_4_ and FeCl_3_. 6H_2_O (0.06 mole) mixed in deionized water was heated to 32 °C followed by drop wise addition of pyrrole (0.02 mole). This mixture was stirred at −2 °C for 24 h. The mixture was centrifuged, washed with distilled water and acetone and dried at 80 °C for 24 h to obtain rGO/MeFe_2_O_4_/Ppy nanocomposite.

### Characterizations

Morphology of GO, rGO/MnFe_2_O_4_, rGO/MnFe_2_O_4_/Ppy, rGO/NiFe_2_O_4_ and rGO/NiFe_2_O_4_/Ppy was analysed by field emission scanning electron microscopy (FESEM, Quanta 450, FEI, USA). Nanocomposites were further investigated by using X-ray diffraction (600 Miniflex, Rigaco, Japan). Fourier Transform Infrared (FTIR) spectroscopy (Nicolet 6700 Thermo Fisher) in transmittance mode and range 400–4000 cm^−1^ was used to identify the functional groups of synthesized nanocomposites. Thermal stability was measured by using a thermal gravimetric analyser (TGA, Q500, TA Instruments, USA) under air where the samples were heated up to 900 °C at a heating rate of 10 °C min^−1^ at RT.

### Electrochemical characterizations

All electrochemical measurements like cyclic voltammetry (CV), galvanostatic charge/discharge (CD) and electrochemical impedance spectroscopy (EIS) were carried out in 1 M H_2_SO_4_ using two electrodes system. Gravimetric and areal capacitances of electrodes were calculated from cyclic voltammetry curves by using equations 3–4, respectively.

3 $$Cs=\frac{4{\int }_{v1}^{v2}idV}{mV{\rm{\Delta }}V}$$

4 $$Cs=\frac{2{\int }_{v1}^{v2}idV}{aV{\rm{\Delta }}V}$$

here ${\int }_{v1}^{v2}idV$ is the integrated area for CV curve, *s* is the scan rate, *V* is *2 × (V*max-*V*min) the potential window, *m* is the mass of single electrode and *a* is the foot-print device area. Specific power and specific energy of electrodes were calculated from cyclic voltammetry by using equations 5–6, respectively.

5 $$P=\frac{{\int }_{v1}^{v2}idV}{m}$$

6 $$E=\frac{{\rm{\Delta }}V{\int }_{v1}^{v2}idV}{3600ms}$$

Electrochemical performance of synthesized nanocomposites was characterized by using CHI760C Electrochemical workstation. Electrode material was fabricated by mixing active material i.e., nanocomposites, carbon black and binder (80:10:10) in 10 mL of ethanol. Polytetrafluoroethylene (60% wt in H_2_O) was used as binder. Mixture was ultrasonicated till completely dispersed, filtered by using filter paper and dried overnight at RT. Supercapacitor was made by using two electrodes of same material with active area of 1 cm^2^. Piece of filter paper dipped in 1 M H_2_SO_4_ was used as separator between two electrodes. Gold electrodes were used as current collectors. Cyclic voltammetry (CV) testing was carried out between 0–1 V at scan rates from 5–2000 mVs^−1^. Charging/discharging (CD) measurements were carried out in voltage window between 0–1 Vat 1–20 Ag^−1^. EIS measurements were done from 0.01 Hz to 100 KHz at an open circuit potential with an AC voltage amplitude of 5 mV. All measurements were carried out at RT.

### Ethical approval and informed consent

The methods used in this work were carried out in accordance with the relevant guidelines and and regulations.

## Conclusions

In summary a simple, scalable and environmentally sustainable method for preparation ternary composite electrodes for supercapacitors applications consisting of rGO, mixed metal doped Iron oxide and conductive Ppy is presented. New method using NaOH with double roles to replace hydrazine for GO reduction and assist the formation of MeFe_2_O_4_ is demonstrated for the first time. This process allows simple one step and scalable synthesis of graphene/metal doped iron oxide (rGO/MeFe_2_O_4_) (Me = Mn, Ni) not possible before. Electrochemical characterizations showed binary rGO/MnFe_2_O_4_composite electrode delivers specific capacitance of 147 Fg^−1^ and areal capacitance of 232 mFcm^−2^ in two electrodes system at scan rate of 5 mVs^−1^. Compared with previous results this is the highest value among all synthesized rGO/MeFe_2_O_4_ electrodes reported in literature. Further significant improvement in performance was observed in ternary composite system after introduction of Ppy showing the capacitances of were increased to 250 Fg^−1^ and 395 mFcm^−2^ for rGO/MnFe_2_O_4_/Ppy electrode. Compared with previous results this is the highest value among all synthesized binary rGO/MeFe_2_O_4_ and ternary rGO/MnFe_2_O_4_/Ppy electrodes reported in literature. These results confirmed that using this simple synthetic strategy it is possible to prepare synergistic composites electrode materials by combination of graphene metal doped iron oxide conductive polymers and promising strategy for designing and tailoring properties of high performing supercapacitors.

## Supplementary information

Supplementary info
