# Supplementary material for: Facile synthesis of ternary graphene nanocomposites with doped metal oxide and conductive polymers as electrode materials for high performance supercapacitors
Source: Sci Rep. 2019 Apr 12;9:5974. doi: 10.1038/s41598-019-41939-y (PMC6461681; doi:10.1038/s41598-019-41939-y)
Supplement: Supplementary file 1 — Supplementary info [file 41598_2019_41939_MOESM1_ESM.docx]

**Supporting information**

**Facile synthesis of ternary** **graphene nanocomposites with doped metal oxide and conductive polymers as electrode materials for high performance supercapacitors**

**Saira Ishaq^1,2,3^, Mahmoud Moussa^2,3^, Farah Kanwal^1^, Muhammad Ehsan^1^, Muhammad Saleem^1^, Truc Ngo Van^2^, Dusan Losic^2,3*^**

**^1^**Institute of Chemistry, University of the Punjab, Lahore 54590, Pakistan

**^2^**School of Chemical Engineering, The University of Adelaide, Adelaide 5005, SA, Australia

**^3^**ARC Research Hub for Graphene Enabled Industry Transformation, The University of Adelaide, Adelaide 5005, SA, Australia

Corresponding author**^*^**

E-mail address: dusan.losic@adelaide.edu.au

**Characterization**

**
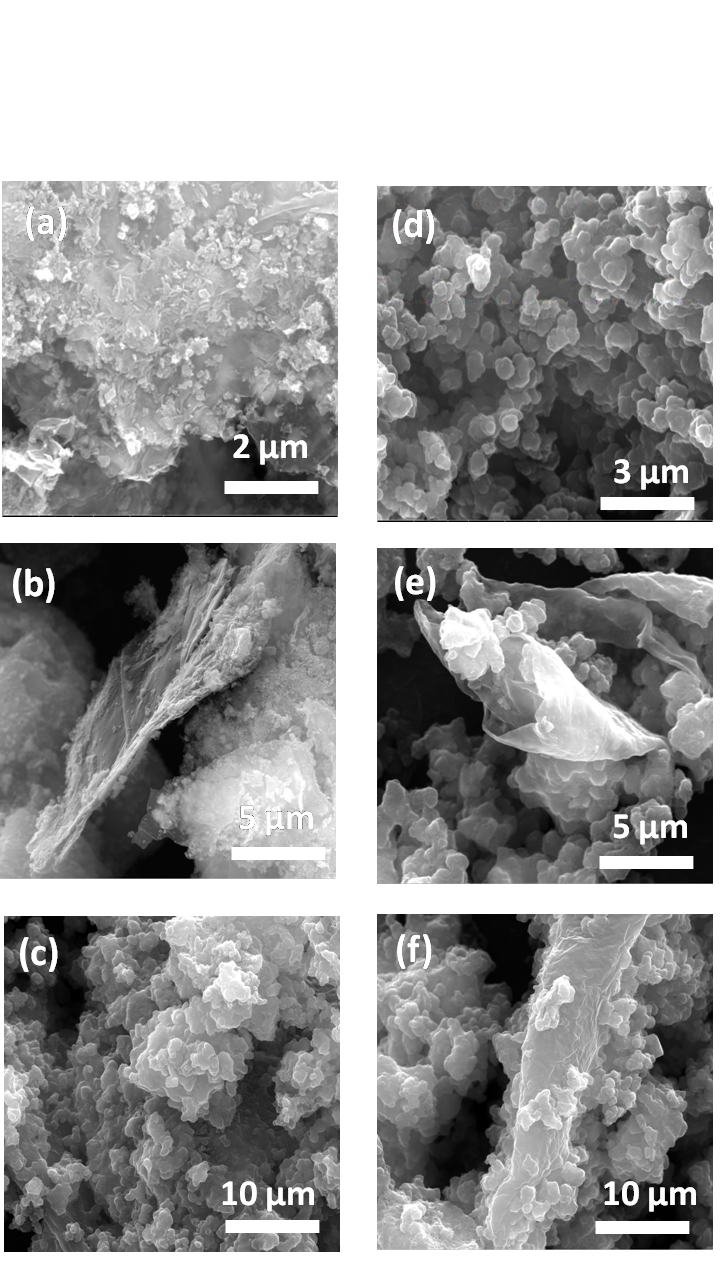
**

Fig. S1. FESEM images of (a-c) rGO/MnFe_2_O_4_ (d-f) rGO/MnFe_2_O_4_/Ppy nanocomposites at different magnifications


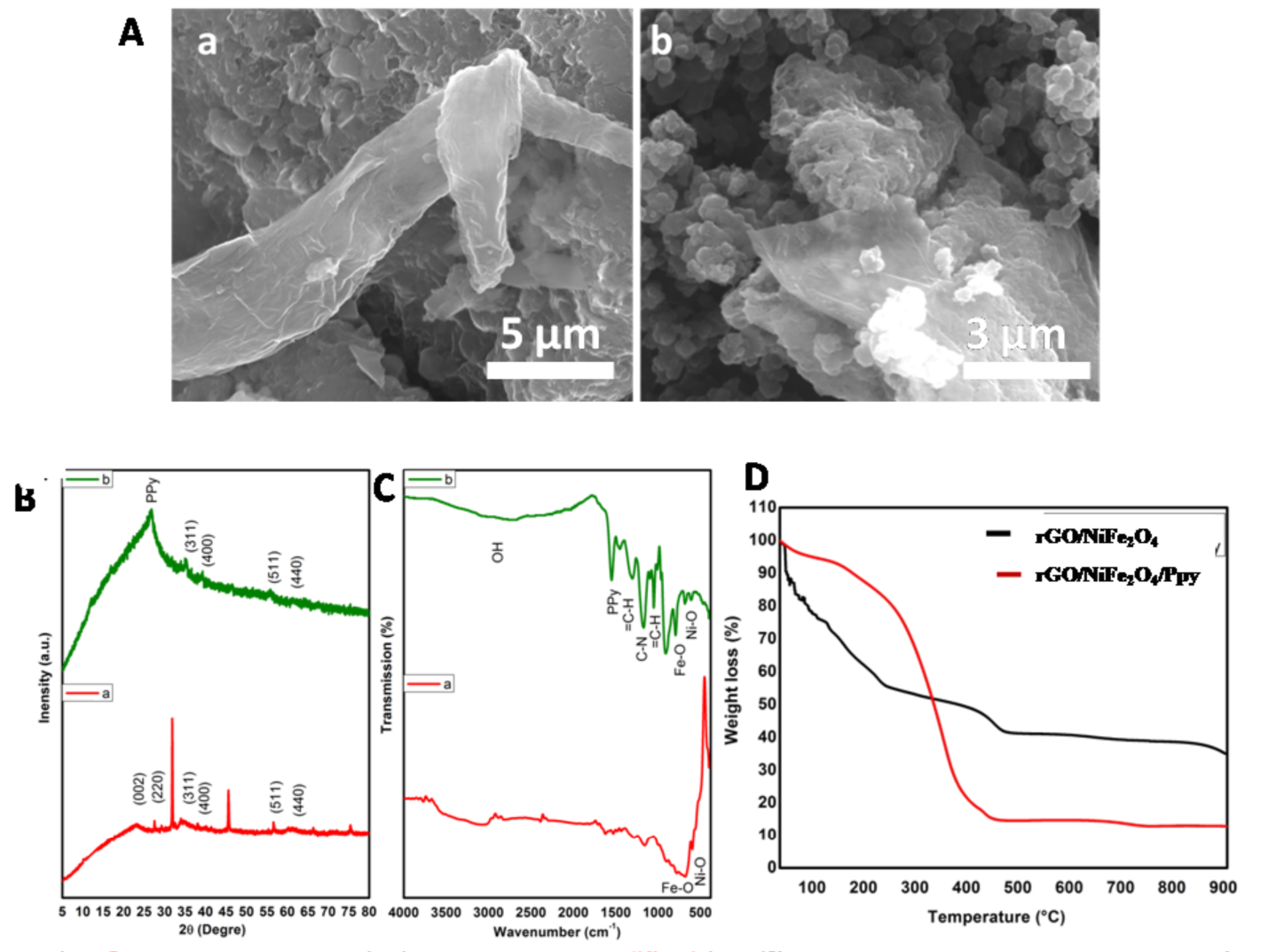


Fig. S2. A) FESEM images B) XRD pattern C) FTIR spectra D) TGA of a) rGO/NiFe_2_O_4_ b) rGO/NiFe_2_O_4_/Ppy nanocomposites

Morphology of rGO/NiFe_2_O_4_ and rGO/NiFe_2_O_4_/Ppy is shown in FESEM images (Fig S2A). NiFe_2_O_4_ nanoparticles are clearly seen distributed on graphene sheets. Average size of NiFe_2_O_4_ nanoparticles was found to be in the range of 60 - 100 nm. However, Ppy is prominent in rGO/NiFe_2_O_4_/Ppy due to its large quantity.

The XRD patterns of as synthesized rGO/NiFe_2_O_4_ and rGO/NiFe_2_O_4_/Ppy are shown in Fig. S2B. XRD pattern of nanocomposites matches to JCPDS card no. 10-0325 and shows all characteristic peaks of NiFe_2_O_4_ i.e., (111), (220), (311), (400), (511) and (440) [^1^](#_ENREF_1). There is no diffraction peak for GO (001) or graphene (002). It is due to reason that due to crystal growth of rGO/NiFe_2_O_4_ regular stacking of graphene sheets is destroyed [^2^](#_ENREF_2). Peaks of as synthesized rGO/NiFe_2_O_4_ are not very sharp that shows amorphous nature of the synthesized nanocomposites. It is in good agreement with the literature [^1^](#_ENREF_1). rGO/NiFe_2_O_4_/Ppy show characteristic peak of Ppy at 26° [^3^](#_ENREF_3).

FTIR spectra of rGO/NiFe_2_O_4_ and rGO/NiFe_2_O_4_/Ppy are shown in Figure S2C. FTIR spectra of both rGO/NiFe_2_O_4_ and rGO/NiFe_2_O_4_/Ppy show peaks of graphene along with peaks of other components thus confirming formation of rGO/NiFe_2_O_4_ and rGO/NiFe_2_O_4_/Ppy nanocomposites. Peaks below 700 cm^-1^ are attributed to metallic particles. Peaks of GO related to oxygen functional groups do not appear showing conversion of GO into rGO during reduction process[^4-7^](#_ENREF_4).

TG curves of rGO/NiFe_2_O_4_ and rGO/NiFe_2_O_4_/Ppy are shown in figure S2D. It shows that rGO/NiFe_2_O_4_ are thermally more stable than rGO/NiFe_2_O_4_/Ppy. rGO/NiFe_2_O_4_/Ppy show more weight loss than rGO/NiFe_2_O_4_ which shows more thermal stability of rGO/NiFe_2_O_4_ than that of rGO/NiFe_2_O_4_/Ppy. This trend is similar in all nanocomposites and is in accordance with results depicted in previous literature. This increase in thermal stability is due to decrease in mobility and thermal vibration of Ppy chains at rGO/MFe_2_O_4_ -Ppy interface[^8^](#_ENREF_8)^,^[^9^](#_ENREF_9).


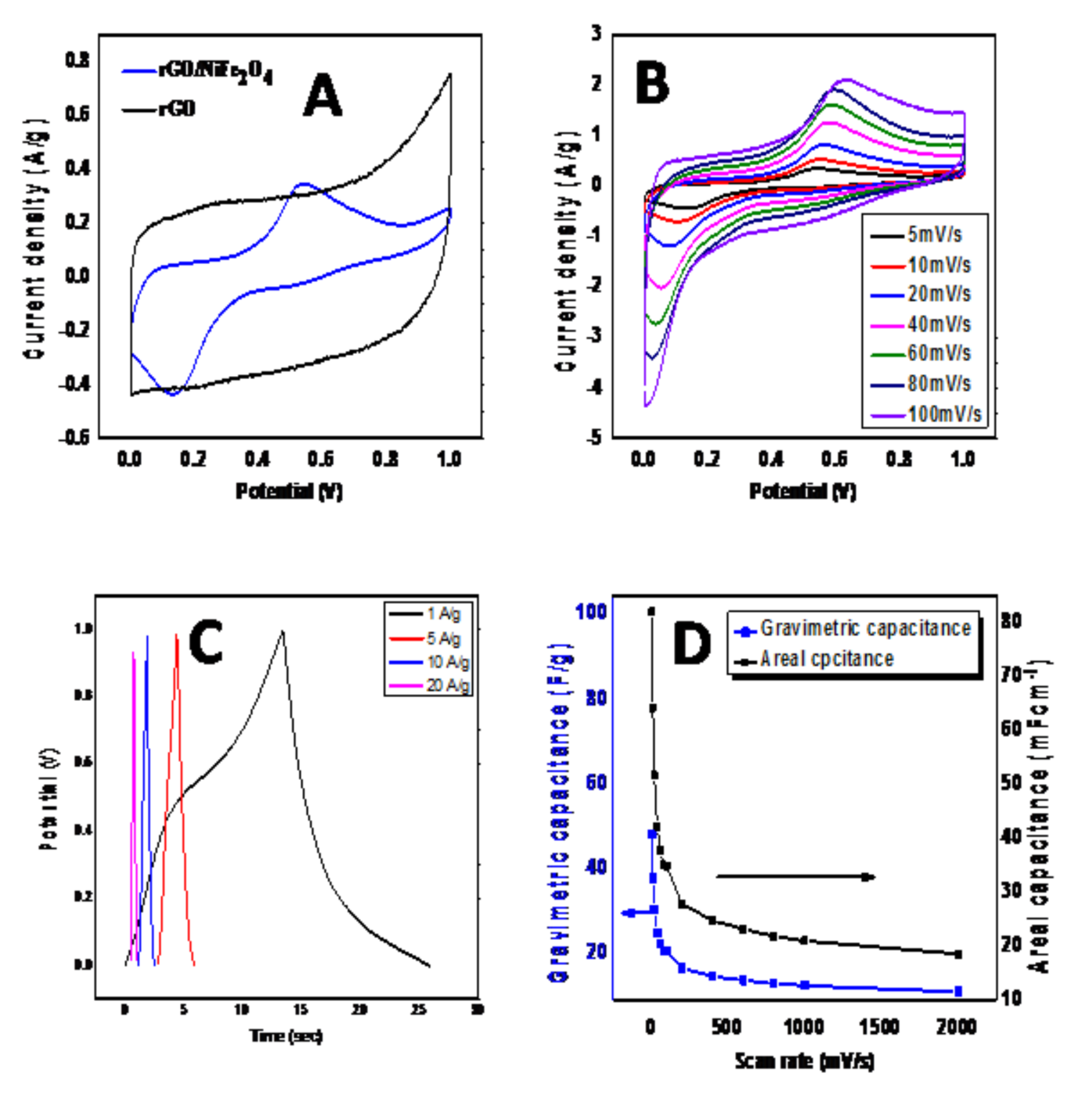


Figure S3. CV curves of A) rGO and rGO/NiFe_2_O_4_ B) CV curves of rGO/NiFe_2_O_4_ nanocomposites at different scan rates (10 – 100 mVs^-1^) C) CD curves of rGO/NiFe_2_O_4_ different current density (1 Ag^-1^, 2 Ag^-1^, 4 Ag^-1^ and 6 Ag^-1^) D) Gravimetric and areal capacitance of rGO/NiFe_2_O_4_ at different scan rates (10 – 2000 mVs^-1^)

Redox peaks appear in CV curves of rGO/NiFe_2_O_4_ and rGO/NiFe_2_O_4_/Ppy electrodes (Figure S3A – S4A) showing their pseudocapacitance behaviour[^10^](#_ENREF_10). Gravimetric capacitance of rGO/NiFe_2_O_4_ and rGO/NiFe_2_O_4_/Ppy electrodes calculated from their CV curves was, 48 Fg^-1^ and 162.9 Fg^-1^ respectively at scan rate of 5 mVs^-1^. At the same scan rate, areal capacitance of rGO/NiFe_2_O_4_ and rGO/NiFe_2_O_4_/Ppy electrodes calculated from their CV curves was 82 mFcm^-2^ and 276.9 mFcm^-1^ respectively. Both gravimetric and areal capacitance of rGO/NiFe_2_O_4_/Ppy was greater than that of rGO/NiFe_2_O_4_, which is attributed to the conducting nature of Ppy.


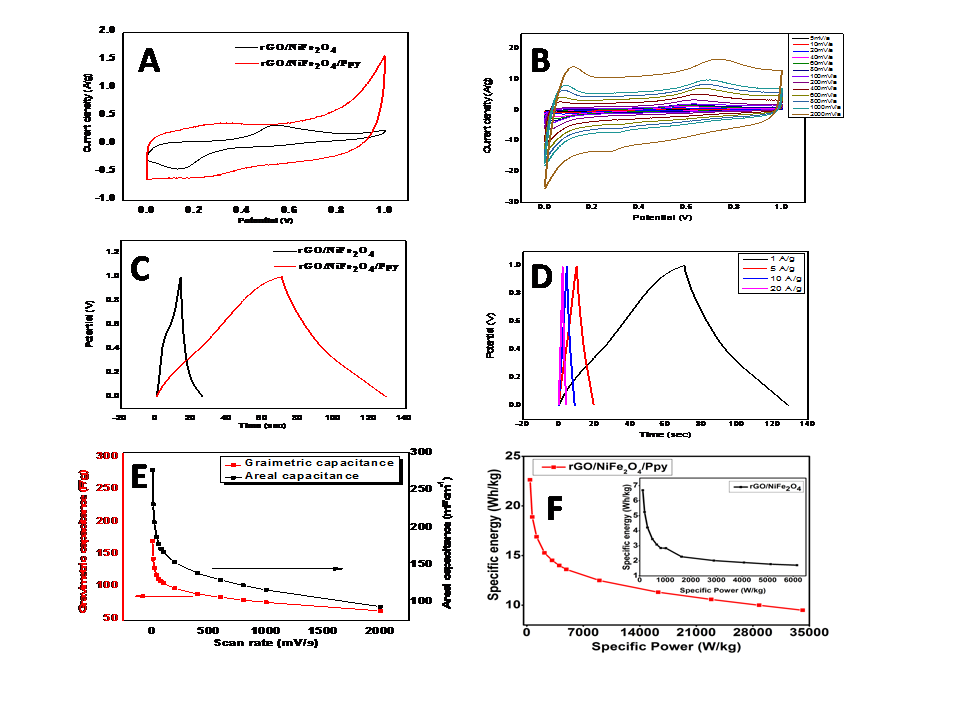


**Figure S4.** A) CV curves of rGO/NiFe_2_O_4_ and rGO/NiFe_2_O_4_/Ppy at 5 mVs^-1^ B) CV curves of rGO/NiFe_2_O_4_/Ppy nanocomposite at different scan rates (10 – 100 mVs^-1^) C) CD curves of rGO/Ni/Fe_2_O_4_ and G/NiFe_2_O_4_/Ppy at 1Ag^-1^ D) CD curves of rGO/NiFe_2_O_4_/Ppy at different current density E) Gravimetric and Areal capacitance of rGO/Ni/Fe_2_O_4_/Ppy at different scan rates (5 – 2000 mVs^-1^) F) Ragon plots of rGO/NiFe_2_O_4_ and rGO/NiFe_2_O_4_/Ppy at different scan rates (5 – 1000 mVs^-1^)

Figure S3B and S4B show the CV curves of rGO/NiFe_2_O_4_ and rGO/NiFe_2_O_4_/Ppy nanocomposites, respectively at different scan rates from 10 - 100 mVs^-1^. Increase in current density with increasing scan rate depicts its fairly good ion response, good EDL capacitance behaviour, more conductivity and low resistance at high scan rate[^10^](#_ENREF_10).

Fig S4C shows galvanostatic charge/discharge (CD) curves of rGO/NiFe_2_O_4_ and rGO/NiFe_2_O_4_/Ppy measured at current density of 1 Ag^-1^. However, CD curves are unsymmetrical showing Faradic behaviour of electrode material. Figure S3C and S4D show the CD curves of the rGO/NiFe_2_O_4_ and rGO/NiFe_2_O_4_/Ppy collected at different current densities. All CD curves are of almost same shape at all current densities, showing that electrode material has ideal capacitive behaviour[^11^](#_ENREF_11).

Figure S3D and S4E represents specific capacitance and areal capacitance of rGO/NiFe_2_O_4_ and rGO/NiFe_2_O_4_/Ppy at various scan rates i.e., 5 – 2000 mVs^-1^. Both gravimetric and areal capacitance decrease with increasing scan rate due to insufficient time available for diffusion of ions and voltage loss [^12^](#_ENREF_12). However at all scan rates increase of specific and areal capacitance (Cs) is in order rGO/NiFe_2_O_4_ < rG/NiFe_2_O_4_/Ppy.

Figure S4F show Ragon plots of rGO/NiFe_2_O_4_ and rGO/NiFe_2_O_4_/Ppy at various scan rates i.e., 5 – 1000 mVs^-1^. At 5 mVs^-1^ specific powers of rGO/NiFe_2_O_4_ and rGO/NiFe_2_O_4_/Ppy are 120.5 Wkg^-1^ and 407.3 Wkg^-1^, respectively which is increased to 6144 Wkg^-1^ and 31468 Wkg^-1^ at 1000 mVs^-1^. While specific energy values of rGO/NiFe_2_O_4_ and rGO/NiFe_2_O_4_/Ppy are 6.7 and 22.6 Whkg^-1^, respectively at 5 mVs^-1^. It decreases to 1.7 Whkg^-1^ and 9.5 Whkg^-1^, respectively. Increases in specific energy from rGO/NiFe_2_O_4_ to rGO/NiFe_2_O_4_/Ppy is due to increased electrical conductivity due to addition of Ppy in rGO/NiFe_2_O_4_/Ppy [^13^](#_ENREF_13)


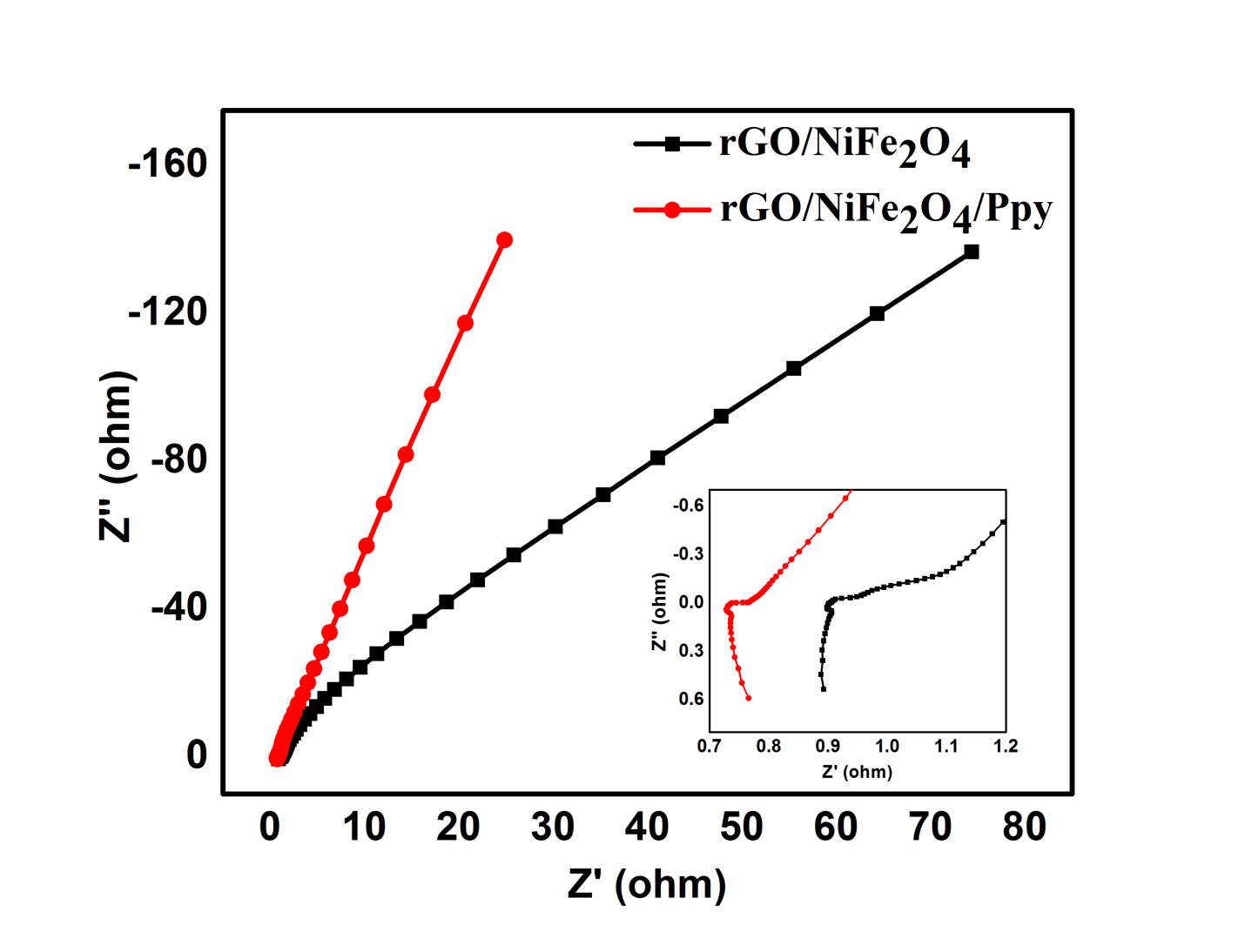


**Figure S5.** Nyquist plots of rGO/NiFe_2_O_4_ and rGO/NiFe_2_O_4_/Ppy

Electrochemical Impedance spectroscopy (EIS) has been performed by two electrodes system in 1 M H_2_SO_4_ at the excited potential 5 mV between frequency range 0.01 Hz to 100 kHz in the form of Nyquist plot shown in Figure S5. Nyquist plot reveals that rGO/NiFe_2_O_4_ and rGO/NiFe_2_O_4_/Ppy show very small semicircle at high frequency region, which reveals to charge transfer resistance and solution resistance while a straight line in high frequency region reveals to Warburg resistance. rGO/NiFe_2_O_4_/Ppy showed small equivalent series resistance of 0.77 Ω while rGO/NiFe_2_O_4_ showed greater resistance of 0.89 Ω. However, in low frequency region, rGO/NiFe_2_O_4_/Ppy has more slope as compare to slope of rGO/NiFe_2_O_4_ explaining that former has better capacitance than later that is consistent with results of CV and CD.

**References**

1 Maensiri, S., Masingboon, C., Boonchom, B. & Seraphin, S. A simple route to synthesize nickel ferrite (NiFe_2_O_4_) nanoparticles using egg white. *Scripta Materialia* **56**, 797-800 (2007).

2 Yan, J. *et al.* Advanced asymmetric supercapacitors based on Ni(OH)_2_/graphene and porous graphene electrodes with high energy density. *Advanced Functional Materials* **22**, 2632-2641 (2012).

3 Bose, S. *et al.* In-situ synthesis and characterization of electrically conductive polypyrrole/graphene nanocomposites. *polymer* **51**, 5921-5928 (2010).

4 Marcano, D. C. *et al.* Improved synthesis of graphene oxide. *ACS nano* **4**, 4806-4814 (2010).

5 Sahoo, B., Sahu, S. K., Nayak, S., Dhara, D. & Pramanik, P. Fabrication of magnetic mesoporous manganese ferrite nanocomposites as efficient catalyst for degradation of dye pollutants. *Catalysis Science & Technology* **2**, 1367-1374 (2012).

6 Gao, L., Yue, W., Tao, S. & Fan, L. Novel strategy for preparation of graphene-Pd, Pt composite, and its enhanced electrocatalytic activity for alcohol oxidation. *Langmuir* **29**, 957-964 (2013).

7 Si, Y. & Samulski, E. T. Synthesis of water soluble graphene. *Nano letters* **8**, 1679-1682 (2008).

8 Wang, Y., Huang, Y., Wang, Q., He, Q. & Chen, L. Preparation and electromagnetic properties of Polyaniline (polypyrrole)-BaFe12O19/Ni0. 8Zn0. 2Fe_2_O_4_ ferrite nanocomposites. *Applied surface science* **259**, 486-493 (2012).

9 Lozano, K. & Barrera, E. Nanofiber‐reinforced thermoplastic composites. I. Thermoanalytical and mechanical analyses. *Journal of Applied Polymer Science* **79**, 125-133 (2001).

10 Xiong, P. *et al.* Ternary manganese ferrite/graphene/polyaniline nanostructure with enhanced electrochemical capacitance performance. *Journal of Power Sources* **266**, 384-392 (2014).

11 Mondal, S., Rana, U. & Malik, S. Graphene quantum dot-doped polyaniline nanofiber as high performance supercapacitor electrode materials. *Chemical Communications* **51**, 12365-12368 (2015).

12 Dubal, D. P. & Holze, R. All-solid-state flexible thin film supercapacitor based on Mn_3_O_4_ stacked nanosheets with gel electrolyte. *Energy* **51**, 407-412 (2013).

13 Iqbal, M. F., Ashiq, M. N., Iqbal, S., Bibi, N. & Parveen, B. High specific capacitance and energy density of synthesized graphene oxide based hierarchical Al_2_S_3_ nanorambutan for supercapacitor applications. *Electrochimica Acta* **246**, 1097-1103 (2017).
